# Acupuncture for perioperative care of total hip arthroplasty: A systemic review of controlled trials and meta-analysis

**DOI:** 10.1097/MD.0000000000015198

**Published:** 2019-04-12

**Authors:** Hee-Ra Shin, Kyungtae Park, Jihye Seo, Sung-Hu An, Seung-Ryong Yeom, Young-Dal Kwon

**Affiliations:** a Department of Korean Medicine Rehabilitation; b Clinical Trial Center, Gwangju Medical Center, College of Korean Medicine, Wonkwang University, Gwangju, Republic of Korea.

**Keywords:** acupuncture, perioperative care, protocol, systematic review, total hip arthroplasty

## Abstract

**Introduction:**

This protocol is intended to conduct a systematic review and meta-analysis to evaluate the efficacy and safety of acupuncture in perioperative care of total hip arthroplasty patients.

**Methods and analysis:**

The following databases will be searched from their inception to November 2018 without language restrictions: MEDLINE, EMBASE, the Cochrane Central Register of Controlled Trials, the Cumulative Index to Nursing and Allied Health Literature, Oriental Medicine Advanced Searching Integrated System, Korean Traditional Knowledge Portal, KoreaMed, DBPIA, Research Information Service System, including the China National Knowledge Infrastructure. Any randomized controlled trials related to perioperative care of total hip arthroplasty will be included. The primary outcomes of this study are dosage of analgesics and pain intensity. For secondary outcomes, Harris hip score, incidence of postoperative cognitive dysfunction, mini-mental state exam score, incidence of deep vein thrombosis, D-dimer and fibrinogen level, adverse events will be assessed. Data analysis and synthesis will be carried out using RevMan version 5.3. The methodological quality will be assessed by the Cochrane Collaboration risk of bias tool.

**Systematic review registration:**

PROSPERO CRD42018112123.

## 1 Introduction

Globally, total hip arthroplasty (THA) is a very common surgery in orthopedics. This is considered a successful, safe and cost-effective medical intervention to restore functionality of the hip joint and to regain pain-free mobility in patients suffering from severe joint disease or trauma.^[1]^

However, despite the advantages of THA and the advances in surgical technique and implant materials, numerous complications such as postoperative pain, joint dysfunction, postoperative cognitive dysfunction (POCD) and venous thrombosis still remain a concern.^[2–5]^

Moreover, systemic opioid analgesics, commonly used to reduce severe pain during or after major surgery, can cause various side effects such as respiratory depression, decreased intestinal motility, nausea, vomiting, and itching, which can adversely affect the quality of patient's life and may lead to significant morbidity. And even in some cases, it is difficult to achieve sufficient pain relief effect by opioid analgesics alone.^[6–8]^ Since there are many difficulties and limitations in caring patients undergoing THA, studies on alternative and multilateral ways of intervention are needed.

Acupuncture is one of the Korean medicine treatments inserting sterile needles into certain anatomical locations so-called “meridian” on the body surface. It is a medical intervention that prevents, mitigates, and cures diseases through the physical stimuli eliciting neurohormonal responses of the body system.^[9]^

A lot of studies support the effectiveness of acupuncture. It is most often used for pain relief, and also for a wide range of other clinical conditions.^[10–12]^

And these days, a variety of clinical studies have been conducted on patients undergoing hip arthroplasty. And acupuncture is found to be effective in the management of patient's complications such as postoperative pain and hip joint dysfunction, POCD and venous thrombosis.^[13–15]^

However, to our knowledge, there has been no systematic review regarding acupuncture for perioperative care of patients undergoing THA. So in this review, systematic review will be implemented to assess the efficacy and safety of acupuncture treatments associated with THA in the aforementioned 4 aspects. Meta-analysis will also be conducted if data synthesis is possible.

## 2 Methods and analysis

This protocol is based on Preferred Reporting Items for Systematic Review and Meta-Analysis Protocols (PRISMA-P) guidelines and the Cochrane Handbook for Systematic Reviews of Interventions.^[16]^ The protocol for this systematic review has been registered in the International Prospective Register of Systematic Reviews (PROSPERO) with registration number: PROSPERO CRD42018112123. We will conduct a systematic review on the basis of this protocol, but if there is any amendment, it will be tracked in PROSPERO.

### 2.1 Data sources and search strategy

We will search the following databases from their inception to November 2018, without language restrictions. MEDLINE, EMBASE, the Cochrane Central Register of Controlled Trials, the Cumulative Index to Nursing and Allied Health Literature, Oriental Medicine Advanced Searching Integrated System, Korean Traditional Knowledge Portal, KoreaMed, DBPIA, Research Information Service System, including the China National Knowledge Infrastructure. We will also search the reference lists of the relevant articles and conduct a manual search on google scholar to review additional trials. Not only the published literature in journals but also “gray literature” such as theses and conference publications will be included. We will organize search terms into terms for patient conditions, terms for intervention, terms for study design. The example of search strategy for MEDLINE is shown in Table 1.

**Table 1:**
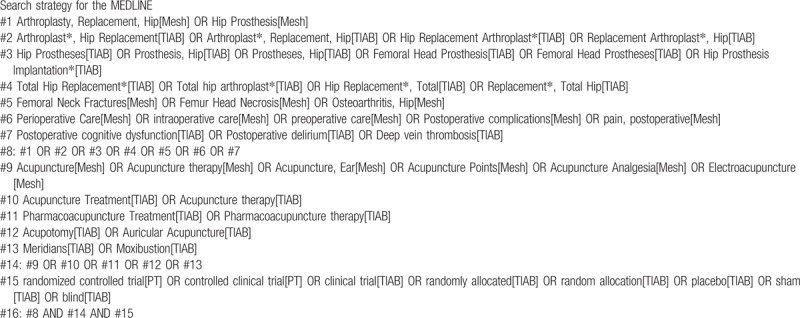
Search strategy for the MEDLINE.

### 2.2 Inclusion criteria

#### 2.2.1 Types of participants

Among the hip arthroplasty surgery, hemiarthroplasty (HA) will be excluded, and only patients undergoing THA will be included. In case of THA, we will not restrict the surgical approach and type. And we will not restrict eligible patients depending on the cause of the operation, age, or gender.

#### 2.2.2 Types of interventions

Any type of needling procedure with stimulating certain points will be included in acupuncture therapy, such as manual acupuncture, electro-acupuncture, auricular acupuncture, Wrist-ankle acupuncture or dry needling on tender points. Acupuncture treatment alone or as an adjunct to other treatments will be included. In case of combination with other treatments, only the study that control group treated the same as the experimental group will be included. Control interventions will include no treatment, other conventional treatment (including analgesics, manual therapy, usual care, and so forth.), or sham/placebo interventions.

#### 2.2.3 Types of outcome measures

The primary outcomes are as follows:

- Pain intensity as assessed by validated measurement scales such as visual analog scale and numerical rating scale or specific pain scale.
- Dosage of analgesics.

The secondary outcomes are as follows:

- Functional evaluation of hip joint; Harris hip score.
- Incidence of POCD.
- Mini-mental state exam score.
- D-dimer and fibrinogen level.
- Incidence of deep vein thrombosis.
- Adverse event of analgesics.
- Adverse event of acupuncture.

#### 2.2.4 Type of studies

Only randomized controlled trials (RCT) will be included. Quasi-RCT using inappropriate random sequence generation methods such as allocation by birthdate, visit date or alternate method will be excluded. In case of only the expression “randomization, randomly divided” is stated without the specific randomization methods, it will be included in this review. But the related risk of bias will be evaluated carefully. Other study designs such as in vivo, in vitro, case reports, and retrospective studies will be excluded.

### 2.3 Study selection

Study selection process will be conducted by 2 independent authors (HRS and KP) according to the above criteria. Searched results will be exported to the Endnote referencing program and duplicate studies will be removed. Then, we will screen and evaluate the titles and abstracts to select the studies that are relevant to our review. After that, we will evaluate the full texts of the selected studies for eligibility. Any disagreements about study selection will be solved through discussion with other researchers. The process of study selection will be documented and summarized in accordance with the PRISMA guidelines and flow chart, and also the reason for excluded studies will be provided.

### 2.4 Data extraction

Two independent authors will perform data extraction and crosscheck the results. We will use a standardized data extraction form (Excel, Microsoft, 2010) agreed by all the authors. When there are any discrepancies or disagreements, we will solve the problem through discussion with other authors.

The extracted data will include the first author, year of publication, country, patient characteristics, details about surgery, cause of the surgery, sample size and dropouts, intervention and comparison details, outcomes and adverse events.

### 2.5 Assessment with risk of bias and quality

The quality assessment of included studies will be conducted by 2 independent authors (HRS and KP) using the Cochrane Collaboration risk of bias tool. Random sequence generation, allocations concealment, blinding of the participants and personnel, blinding of the outcome assessments, incomplete outcome data, selective reporting, and other sources of bias will be assessed for each included studies. Each domain will be categorized into 3 groups; “high risk,” “low risk,” or “unclear.” Evaluation results will be recorded in Excel (Microsoft, 2010) form and shared with researchers. Any disagreements will be solved by discussion with other authors.

We will use the Grading of Recommendations Assessment, Development, and Evaluation approach to evaluate the quality of evidence for our review.

### 2.6 Data synthesis and analysis

The data synthesis and analysis will be conducted using Review Manager version 5.3 software (Cochrane) for windows. Analysis of the participant characteristics, interventions, and outcomes will be conducted for all eligible studies. A quantitative synthesis will be performed if studies of the same type of intervention, comparison, and outcome measures exist. For dichotomous data, we will present the outcomes as relative risks with 95% confidence intervals (CIs). For continuous data, the data will be pooled as the mean difference or standardized mean difference with 95% CIs. If there are any insufficient data, we will contact the authors to request insufficient information. If data synthesis is impossible, we will conduct narrative analysis.

Heterogeneity of effect size between selected studies will be assessed by chi-squared test and *I*^2^ statistic. When *I*^2^ values are calculated more than 50% (*I*^2^ >50%), we will consider it has medium heterogeneity. And when *I*^2^ values are calculated more than 75% (*I*^2^ >75%), we will consider it has significant heterogeneity. In the meta-analyses, we will choose a random effect model when heterogeneity is significant (*I*^2^ >75%), whereas a fixed-effect model will be considered when heterogeneity is not significant. And a fixed-effect model will be considered when the number of studies included in the meta-analysis is very small, because between-study variance cannot be estimated.^[17]^ When the heterogeneity is too high to synthesize the data (*I*^2^ >75%), a subgroup analysis will be conducted as follows to investigate the cause of heterogeneity.

### 2.7 Subgroup analysis

In case of significant heterogeneity, we will conduct a subgroup analysis to account for the cause of heterogeneity. The criteria for subgroup analysis are as follows: age, patient's physiological status categorized by American Society of Anesthesiologists score; operation method of acupuncture; surgical approach; cause of the surgery.

### 2.8 Assessment of reporting bias

When there are more than 10 studies included in the analysis, we will assess the publication bias by using funnel plots. If asymmetry of the funnel plot is observed, we will consider about the possible reasons.

## 3 Ethics and dissemination

Our review will be disseminated by the publication of a manuscript in a peer-reviewed journal or by presenting it at the relevant academic conference. Since our systematic review will not use the individual patient data, ethical approval will not necessary.

## 4 Discussion

There are 2 types of hip arthroplasty surgery, HA and THA. Since there are different indications between 2 surgery,^[18]^ the prognosis and outcomes of the patients may vary depending on which surgery is taken. Therefore, only patients undergoing THA will be included in this review.

Although hip arthroplasty provides satisfactory results for patients with hip joint disease with the advances of surgical techniques and surgical materials, there are still many problems associated with surgery. Problems such as difficulty in controlling pain during or after the surgery, abuse of opioid analgesics, POCD, and venous thrombosis can exacerbate functional recovery and quality of life or even increase mortality.^[19–22]^ Even the use of anticoagulant and other alternative analgesics can reduce the side effects, not every patient is completely satisfied.^[23,24]^ So the development of non-pharmacologic and alternative interventions is necessary.

Acupuncture has been proved its efficacy and safety in pain control and treatment for other various clinical symptoms through a lot of clinical researches. And these days many clinical studies related to acupuncture treatment for perioperative care of hip arthroplasty are being tried. However, a systematic review and meta-analysis on this issue have not been performed to date. So we developed this protocol to search and analyze the related data, and we will evaluate the efficacy and safety of acupuncture treatment for perioperative care of THA objectively. We expect that the result of our review will provide another effective treatment strategy for patients undergoing THA.

## Author contributions

HRS conceptualized and designed this protocol and drafted the manuscript.

KP registered this protocol, collected and analyzed the data.

JS designed the methodology of this protocol.

SHA collected and analyzed the data, examined the criteria in clinical practice.

SRY reviewed and edited this protocol, examined the criteria in clinical practice.

YDK reviewed and edited this protocol, supervised all of the procedure.

All authors have read the final manuscript and approved it to be submitted.

**Conceptualization:** Hee-Ra Shin, Young-Dal Kwon.

**Data curation:** Kyungtae Park.

**Investigation:** Kyungtae Park, Sung-Hu An.

**Methodology:** Jihye Seo, Seung-Ryong Yeom, Young-Dal Kwon.

**Supervision:** Seung-Ryong Yeom, Young-Dal Kwon.

**Writing – original draft:** Hee-Ra Shin.

**Writing – review and editing:** Kyungtae Park, Jihye Seo, Sung-Hu An, Seung-Ryong Yeom, Young-Dal Kwon.

Hee-Ra Shin orcid: 0000-0002-4041-2267.
